# An Overview of the Role of Furin in Type 2 Diabetes

**DOI:** 10.3390/cells12192407

**Published:** 2023-10-05

**Authors:** Sulaiman K. Marafie, Fahd Al-Mulla

**Affiliations:** 1 Biochemistry and Molecular Biology Department, Dasman Diabetes Institute, P.O. Box 1180, Dasman 15462, Kuwait; 2 Genetics and Bioinformatics Department, Dasman Diabetes Institute, P.O. Box 1180, Dasman 15462, Kuwait

**Keywords:** furin, type 2 diabetes, prediabetes, mTOR, proprotein convertase, β-cell, furin variants, metabolic disorders, therapeutic target

## Abstract

Post-translational modifications (PTMs) play important roles in regulating several human diseases, like cancer, neurodegenerative disorders, and metabolic disorders. Investigating PTMs’ contribution to protein functions is critical for modern biology and medicine. Proprotein convertases (PCs) are irreversible post-translational modifiers that have been extensively studied and are considered as key targets for novel therapeutics. They cleave proteins at specific sites causing conformational changes affecting their functions. Furin is considered as a PC model in regulating growth factors and is involved in regulating many pro-proteins. The mammalian target of the rapamycin (mTOR) signaling pathway is another key player in regulating cellular processes and its dysregulation is linked to several diseases including type 2 diabetes (T2D). The role of furin in the context of diabetes has been rarely explored and is currently lacking. Moreover, furin variants have altered activity that could have implications on overall health. In this review, we aim to highlight the role of furin in T2D in relation to mTOR signaling. We will also address furin genetic variants and their potential effect on T2D and β-cell functions. Understanding the role of furin in prediabetes and dissecting it from other confounding factors like obesity is crucial for future therapeutic interventions in metabolic disorders.

## 1. Introduction

Post-translational modifications (PTMs) are chemical or enzymatic alterations that occur on proteins after they are synthesized from their corresponding genes. They are essential for protein function and are involved in various biological processes, including protein folding, localization, stability, activity, and interaction with other proteins. Common types of PTMs include phosphorylation, glycosylation, acetylation, methylation, ubiquitination, and proteolytic cleavage. Moreover, PTMs are highly dynamic and can either be reversible or irreversible; they can also have profound effects on protein functions and cellular processes. The dysregulation of PTMs has been implicated in several human diseases, including cancer, neurodegenerative disorders, and metabolic disorders. Therefore, understanding the role of PTMs in protein function during health and disease is a crucial area of research in modern biology and medicine [1,2]. One important example of an irreversible PTM is the cleavage of proteins by proteases at specific sites or motifs. Such cleavage causes conformational changes to proteins, affecting their downstream biological functions. Among those proteolytic enzymes are a family of serine proteases, known as proprotein convertases (PCs), that have been extensively studied owing to their robust involvement in various biological processes and have been considered as key targets for novel therapeutics [3]. Compared to the other members of the PC family, furin has been extensively studied as a convertase model. Therefore, in this review, we aim to highlight the role of furin in type 2 diabetes (T2D) in relation to the well-established mammalian target of the rapamycin (mTOR) signaling pathway. Illuminating the relationships between mTOR and furin is vital for future therapeutic interventions in metabolic disorders. We will also discuss different variants of furin and their potential effect on T2D and β-cell functions, which is currently lacking in the literature.

## 2. PCs Are Key Regulators of Signaling Pathways

Proteolytic cleavage downstream of the endoplasmic reticulum is one of the key steps to biologically ensure the activity of several growth factors. PCs are activated enzymes that cleave their target proteins regulating different cellular pathways. They are initially synthesized as inactive zymogens that undergo autocatalytic activation in the trans-Golgi network. Once active, PCs recognize and cleave specific target proteins at the C-terminal region containing the R-X-R motif generating biologically active fragments. In turn, the proteolytic cleavage can either activate or inactivate its target protein(s) regulating different cellular processes. The activity of such proteases is tightly controlled ensuring precise and regulated proteolysis. Additionally, PCs are important calcium-dependent processing enzymes that act on such motifs to promote the activity of many of their downstream targets. To date, nine mammalian PCs were identified that belong to the family of kexin/subtilisin-like proteins. These include furin, PC1 (also known as PC3), PC4, PACE4 (Paired Basic Amino Acid Cleaving Enzyme 4), PC5 isozymes A and B (also known as PC6), PC7, SKI-1/S1P (Site-1 Protease), and PCSK9 (Proprotein Convertase Subtilisin/Kexin Type 9) [4,5]. Some PCs have overlapping tissue distributions whereas others have more specific ones.

PC1 is primarily expressed in endocrine tissues such as the pituitary and pancreatic islet cells. It plays a significant role in converting and processing prohormones like insulin and glucagon into their active forms. PC2, on the other hand, is mainly expressed in neuronal tissues and is involved in the processing of prohormones in the central nervous system. For example, it is crucial for the maturation of pro-opiomelanocortin (POMC) into adrenocorticotropic hormone (ACTH) and other biologically active peptides [4,6,7]. PC4 is less studied compared to the other PC members and is exclusively found in testicular germ cells [8]. Like PC2, PACE4 is also involved in the processing of prohormones but is expressed in various tissues including the brain, placenta, and reproductive organs [9,10]. Furin, PC5, and PC7 are also ubiquitously expressed [4] and promote the activity of many substrates, including growth factors, such as endothelin-1 [11] and transforming growth factor (TGF)-b1 [12]; extracellular matrix proteins [13] and matrix metalloproteases [14]; viral envelope glycoproteins [13]; and adhesion molecules [14]. SKI-1/S1P is an example of an endoplasmic reticulum-specific protease that acts on membrane-bound transcription factors and enzymes such as sterol regulatory element-binding proteins (SREBPs) and plays a role in regulating lipid metabolism [15,16]. Lastly, PCSK9 mainly functions in regulating cholesterol metabolism by mediating the degradation of the low-density lipoprotein receptor (LDLR) [17].

Despite some PCs having overlapping tissue distribution, they exert diverse functions and can regulate or be regulated differentially. For instance, studies have shown that PC5 is regulated by platelet-derived growth factor (PDGF) via the phosphatidylinositol-3-kinase/p70 ribosomal protein S6 kinase (PI3K/p70^S6^) signaling pathway in vascular smooth muscle cells. This was demonstrated using wortmannin and rapamycin, inhibitors of the PI3K and mammalian target of rapamycin (mTOR) signaling pathways, respectively, where levels of PDGF-induced PC5 were reduced. However, no effects were observed on other PCs, such as furin or PC7 [18]. Moreover, others have shown that in vascular endothelial cells under stress conditions, furin levels increase while PC5 expression remains unaffected [19]. It is also key to point out the importance of PC redundancies as a compensatory mechanism in the absence of its members to prevent the disruption of crucial physiological processes in cells.

## 3. Furin Is a Convertase Model in Regulating Growth Factors

Furin is an extensively studied factor because of its involvement in regulating many pro-proteins, such as bone morphogenetic protein 4 (BMP4) [20,21], pro-b-nerve growth factor (NGF) [22], insulin receptor (IR) [23], Notch1 receptor [24], and several others, in a calcium-dependent manner. As a result, furin has been linked to several human diseases such as cancers, neurological disorders, and cardiovascular diseases. In cancer, furin has been shown to promote tumor growth and invasiveness due to its overexpression or activation of key growth factors and cytokines involved in cancer progression [25]. Moreover, furin has also been associated with neurodegenerative disorders like Alzheimer’s disease where its increased activity contributes to the accumulation of neurotoxic peptides in the brain [26]. In the context of cardiovascular diseases, furin enhances the activation of proteins involved in regulating blood pressure and lipid metabolism. As a result, dysregulation of such processes leads to complications such as hypertension and atherosclerosis in a furin-mediated manner [27,28]. More recently, furin attracted significant attention during the coronavirus disease (COVID-19) pandemic as it was implicated in affecting the aggressiveness of severe acute respiratory syndrome coronavirus-2 (SARS-CoV-2) and predicting disease severity. Specifically, seven deleterious furin variants were detected in the study cohort, indicating a possible decrease in its protease function, which could potentially reduce the risk of SARS-CoV-2 infection. Moreover, the furin variants were significantly associated with hypertension, one of the common outcomes associated with the increased risk of SARS-CoV-2 infection [29]. It has been recently demonstrated that furin is a protease that promotes the processing of the spike (S) protein of SARS-CoV-2. The S protein is important for viral entry into host cells and contains a furin functional cleavage site. Once the site has been cleaved, two subunits are formed, namely S1 and S2. The S1 subunit contains the receptor-binding domain that allows the virus to attach to its host cell, whereas the second S2 subunit facilitates viral–host cell membrane fusion promoting viral entry. As a result, the presence of a furin cleavage site could be attributed to the increased infectivity and transmissibility of SARS-CoV-2 [30,31,32]. Therefore, the development of furin antiviral drugs is of significant importance due to furin’s critical involvement in the viral infection process. The presence of such drugs would help in reducing the cleavage of the viral envelope, which in turn, would reduce viral infectivity and transmission, and more crucially, promote the intervention of diseases such as COVID-19.

Furin is encoded by the *pcsk3* gene and is ubiquitously expressed in many tissues in variable amounts [33,34,35]. However, it is predominantly located in the trans-Golgi network and can shuttle between the cell membrane and endosomes [36]. Early studies on furin performed on rat gastric mucosa cells demonstrated that it is regulated by epidermal growth factor (EGFR) signaling and that its expression is associated with increased levels of TGF upon stimulation [37]. It was later shown that furin is the main converting enzyme for TGF-b1, targeting its unique R-H-R-R motif. Although other PCs are capable of cleaving TGF-b1, only furin is able to block 80% of TGF-b1 processing, both in vitro and in vivo [12]. This was an important finding because TGF- b1 is the most extensively studied isoform of the TGF family and is involved in key biological processes regulating immune suppression and inflammatory reactions [38,39]. Other studies have further emphasized the importance of furin: unlike the case for other PCs such as PC2 and PC4, knocking out mouse furin was embryonically lethal during the early stages of development [40]. Such consequences could be due to the accumulation of several developmental defects that fall in line with the role of furin in the activation and/or maturation of the TGF-b family members, along with several of its other target substrates.

## 4. Furin Regulates Insulin Signaling and Is Associated with Diabetes

### 4.1. Furin Catalyzes the Processing of Insulin Signaling Proteins

T2D is a chronic metabolic disorder characterized by high levels of glucose in the blood. It is caused by a combination of genetic and lifestyle factors, including obesity, physical inactivity, and poor dietary habits. As a result, the body becomes resistant to the effects of insulin and this results in glucose accumulation in the bloodstream, leading to hyperglycemia. Over a prolonged period, chronic hyperglycemia can cause damage to various organs and tissues, including the eyes, kidneys, nerves, and blood vessels [41,42]. There has been growing interest in the role of furin in the development of T2D. One way in which furin may contribute to the development of T2D is through its effect on the processing of proinsulin, a precursor to insulin. Early studies on furin have shown that it is a key catalyst in the production of mature insulin in vitro. It does so by cleaving proinsulin and promoting insulin to its active form [43,44]. In addition to its effect on insulin processing, furin has also been implicated in the processing of other proteins that are involved in glucose regulation and insulin signaling. For example, furin can cleave and activate proglucagon (PG), an endocrine precursor protein that plays a role in glucose regulation and production. Dey and colleagues demonstrated that the processing of PG was mediated by PCs, including furin. The proteolytic cleavage begins at the interdomain site of PG which leads to the generation of two major peptides, namely glicentin and major PG fragment (MPGF), that sets the stage for the upcoming steps of PG processing. Specifically, furin assisted the processing of intestinal MPGF to produce two key regulators of obesity and T2D: glucagon-like peptide 1 (GLP-1) and GLP-2 [45]. More recently, studies have shown that furin also plays a role in the maturation of the IR. This was demonstrated by Zhang et al., where the researchers illustrated furin’s interaction and cleavage of pro-IR, thereby promoting it to its active form. Such an interaction is hindered by the presence of high levels of homocysteine (Hcy) that decrease the levels of IR, leading to insulin resistance, a hallmark of T2D. Moreover, knocking down furin also resulted in the accumulation of pro-IR, emphasizing the crucial role furin plays in regulating insulin resistance [46].

### 4.2. The Association of Furin Levels with Diabetes and Mortality

In addition to furin’s role in assuring the maturation of insulin and PG, others have investigated its association with metabolic diseases, including T2D. For instance, decreased serum levels of triglycerides and increased levels of high-density lipoprotein cholesterol (HDL-C) were associated with polymorphism in the *furin* gene [47]. Elevated furin serum levels were also associated with key hallmarks of metabolic syndrome and diabetes, such as body mass index (BMI) and triglyceride levels [48]. More recently, Fernandez et al. were the first to report an association between elevated fasting plasma furin levels and an increased incidence of diabetes and risk of mortality. The study was conducted on individuals from the population-based Malmö Diet and Cancer Study where participants had undergone a full medical history check as well as physical and laboratory assessments at the baseline. The study found a positive correlation between serum furin levels and fasting blood glucose (FPG) as well as an association between increased serum furin levels at the baseline and the prevalence of diabetes compared to participants without diabetes. Surprisingly, increased furin levels were also associated with increased metabolic traits like BMI, plasma glucose concentrations, insulin, and low- and high-density lipoprotein-cholesterol (LDL-C and HDL-C, respectively). It is worth noting that a small number of participants had diabetes at the baseline who also had increased furin levels compared to the rest of the cohort without diabetes. The study outcome was considered as a predictive model for the risk of diabetes development and mortality. However, their study had some limitations such as the constraint in the cohort’s BMI range used as well as not knowing whether individuals had T1D or T2D [49]. Moreover, since furin has been implicated in autoimmunity [50], it cannot be ruled out that the observed increase in mortality was due to the individual’s autoimmunity as no data were available from the cohort. Despite these limitations, being a longitudinal study in design as well as following up with their cohort displays the strength of the study.

On the other hand, a cross-sectional study was conducted on a Chinese population where contrasting findings to those of the study from Finland were observed. In this study, there was a negative association between plasma furin levels and the risk of developing diabetes. Their cohort demonstrated lower furin serum levels in individuals with prediabetes and diabetes compared to those with normal FPG. Moreover, increased furin serum was associated with a reduced risk of prediabetes and diabetes and vice versa. However, no significant differences were observed between the prediabetes and diabetes groups. Like the study from Finland, a few individuals had diabetes at the baseline which was also associated with high serum furin levels. The researchers concluded that furin deficiency could be considered as an overall biomarker and/or a risk factor for diabetes rather than a precursor for prediabetes. Like the Malmö Diet and Cancer Study cohort, there was no clear BMI stratification and all individuals included in the study were of a specific Han ethnic background. Additionally, comparing furin levels of all participants, those with lower furin levels were more likely to be older, smokers, and drinkers with increased BMI and FPG compared to those with higher furin levels. [51]. Such discrepancies need to be taken into consideration and additional research needs to be performed on individuals with different ethnicities and backgrounds. In a different study using the same population, He and colleagues suggested an association between the hypermethylation of the *FURIN* promoter, which mostly predicts lower furin protein levels and an increased risk of diabetes incidence [52]. As the study mentioned earlier, reduced serum furin levels were also negatively associated with an increased risk of obesity and hypertension which share many risk factors and molecular mechanisms with diabetes [53,54].

Overall, these studies suggest that furin might have a protective role in metabolic diseases and acts as a promising target for the development of novel therapeutics. A counter suggestion that supports the findings of the Finnish study is that increased furin levels might act as a compensatory mechanism to increase insulin receptor synthesis since furin has been shown to have a role in the maturation of the insulin pro-receptor [55]. Another explanation is that increased furin levels can promote β-cell proliferation and differentiation leading to insulin maturation, a role that has also been previously established [56,57]. Nevertheless, it is crucial to address the importance of furin levels for individuals’ overall health and whether its activity differs in different ethnic backgrounds. Further studies are required to fully elucidate the role of furin in T2D and differentiate it from obesity induced T2D and complications. One way to address the discrepancies mentioned in the above studies is to use Mandelian randomization studies. Mendelian randomization is an analytical method that uses genetic variants as instrumental variables for modifiable risk factors that affect population health. It can overcome a major limitation of evidence from observational studies: unmeasured confounding [58]. Such studies, which are unfortunately lacking, are considered as a more accurate means of assessing the direct involvement of furin in disease causation because it alleviates the influence of the many confounding factors, like obesity in T2D.

## 5. Furin and mTOR Signaling in T2D

mTOR is a protein kinase that plays a crucial role in regulating cell growth and metabolism. It is involved in several cellular processes, such as protein synthesis, autophagy, and lipid synthesis. In addition, the mTOR pathway is a complex network of signaling events that are regulated by several proteins and molecules. Its activation is triggered by a variety of factors, including growth factors, nutrients, and stress. There are two main complexes of mTOR: mTOR Complex 1 (mTORC1) and mTOR Complex 2 (mTORC2); both complexes have distinct compositions, functions, and regulatory mechanisms. For instance, mTORC1 is primarily involved in regulating cell growth, metabolism, and protein synthesis. It is mainly activated by growth factors, nutrients (e.g., amino acids and glucose), and energy status to exert its downstream functions. On the other hand, mTORC2, which is also activated by growth factors but is less sensitive to nutrients and energy status, regulates survival, cellular cytoskeleton, and glucose uptake [59,60,61,62,63,64,65]. Overall, the different mTOR complexes play important roles in regulating a variety of cellular processes and their dysregulation has been linked to several diseases in humans, including cancer, diabetes, and neurological disorders. The role of mTOR signaling in the development of T2D is complex and not fully understood. Studies have shown that the dysregulation of mTOR signaling can lead to insulin resistance and impaired glucose metabolism, which are key hallmarks of T2D, in addition to exacerbating the complications associated with the disease, such as cardiovascular disease and diabetic nephropathy [66,67]. Specifically, mTOR exerts its effects via key substrates like Akt, serum/glucocorticoid-regulated kinase (SGK), and protein kinase C (PKC) that play key roles in regulating lipogenesis, glucose uptake, glycolysis, and cell survival [68,69,70]. Consequently, mTOR dysfunction has been implicated in the development of insulin resistance and diabetes. The insulin receptor substrate 1 (IRS-1) is another key target of mTOR signaling that is associated with insulin resistance and an important regulator of insulin-stimulated glucose metabolism [67].

Despite T2D being extensively studied and the well-established role of mTOR signaling in metabolic diseases, the role of PCs (including furin) in the mTOR signaling pathway has been rarely explored. Although there was a study that has shown an association between the protease activity of furin and mTORC1 signaling, it was in the context of renal cell carcinoma [71]. The interest in investigating the role of furin in T2D is driven by the fact that the *furin* gene is located close to the PRC1 locus, which is linked to an increased susceptibility to T2D [72], and the fact that polymorphism in *furin* has also been linked to metabolic syndrome [47]. Additionally, furin is crucial for the acidification of secretory granules regulating the vacuolar-type ATPase (V-ATPase) proton pump, a critical step in β-cell granule maturation [57]. However, the significance of furin in regulating β-cell function is poorly studied. Recently, Brouwers et al. demonstrated a novel molecular mechanism of furin in regulating glucose levels in β-cells. The researchers showed that an absence of furin in β-cells promotes mTORC1-mediated activation of activating transcription factor 4 (ATF-4), leading to β-cell dysfunction. Since furin is abundantly expressed in pancreatic islets, β-cell furin knockout mice models were generated, in which an increase in blood glucose levels was observed, leading to glucose intolerance. The absence of furin also led to a reduction in β-cell mass and affected granule homeostasis due to the disruption of the V-ATPase pump, which promoted the mTORC1-mediated activation of ATF-4. These findings suggested a novel mechanism, wherein furin played an important role in regulating β-cell mass and function, which, in turn, regulates glucose homeostasis via the mTORC1-ATF4 axis [73]. Such studies are important due to the fact that many PCs (including furin) are less explored in cellular secretory pathways. Furin, specifically, is found in immature secretory granules that promote neuropeptide processing and acts as a pro-growth hormone-releasing hormone [74,75]. The significance of the study conducted by Brouwers et al. shows that furin specifically, and not other PCs, plays a crucial role in β-cell function in vivo. It also allows the identification of further furin physiological substrates that could affect β-cell functions and emphasizes its role with relation to the *PRC1* locus [73]. This allows for a better understanding of the pathophysiology of T2D and targeted therapies development.

## 6. Furin Variants, SARS-CoV-2, and T2D

Recently, several variants of furin were identified to have altered activity, which can have implications in both human health and disease. There is currently a lack of scientific data regarding the relationship between furin variants and T2D. Given the complex nature of diabetes and the multitude of factors that can contribute to its development, it is likely that the relationship between furin variants and T2D is multifactorial and influenced by a range of environmental, genetic, and lifestyle factors. Therefore, our colleagues here at the Dasman Diabetes Institute (DDI) in Kuwait have been looking into the association between genetic variants with metabolic diseases in the Kuwaiti population and their frequencies compared to those of the global population. Researchers here at DDI have also developed a variome database, namely the Kuwait Genetic Variant Database (http://variome.dasmaninstitute.org (accessed on 12 June 2023)), where association studies with different variants (including furin variants) are being conducted. Since furin variant frequencies differ between global populations, studying such variants helps better understand the mechanisms by which furin functions and how it interacts with other proteins. This could be used to develop novel treatments for diseases that are caused by aberrant furin activity, such as cancer and certain viral infections. As mentioned earlier, furin variants have been considered to be predictive markers for COVID-19 outcomes [29,76]. Additionally, particular furin variants, namely rs6224 and rs4702, have been suggested to act as a potential markers of mortality and cardiovascular traits in severe COVID-19 [77]. However, the exact mechanisms by which these variants of furin contribute to cardiovascular disease or diabetes are not fully understood yet. In addition, no functional role of furin was addressed in the above study as its activity levels in their cohort were not measured. On the contrary, our colleagues at DDI have recently measured furin’s activity levels and associated it with increased SARS-CoV-2 entry into cells. Moreover, the furin variant R81C (rs148110342) had a much higher frequency in our Kuwaiti population compared to Europe, Africa, or east Asia where its occurrence is extremely rare [29]. Unpublished data from our group demonstrate that the furin R81C variant is detrimental to its activity and could potentially be associated with reduced mortality in Kuwait. As a result, we believe that the R81C variant could play a protective role and possibly lead to a better outcome with regards to metabolic diseases, including diabetes. Additionally, based on our preliminary findings that are in-line with those of Fernandez et. al., we hypothesize that reduced furin levels are rather protective in T2D; we also think that its increased levels in prediabetes could be a consequence to hyperinsulinemia.

## 7. Conclusions

**Figure 1:**
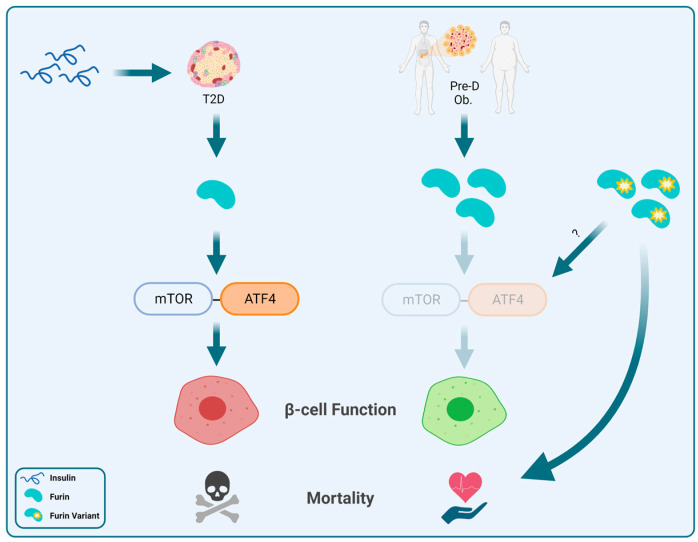
Proposed model of the potential roles of furin and its variants in β-cell function and overall health. T2D, type 2 diabetes; Pre-D, prediabetes; Ob., obesity. (Created with BioRender.com accessed on 12 June 2023).

We aimed for this review to highlight the lack of focus of current research on furin, furin variants, and their potential effect on the development and/or progression of T2D. Figure 1 illustrates a proposed model that summarizes what is currently known regarding furin and what we think is lacking in terms of furin variants’ potential contribution to β-cell function and overall health. Based on the findings mentioned in this review, we hypothesize that individuals with either reduced (left panel) or increased levels (right panel) of furin who also present with prediabetes, T2D, or obesity have affected β-cell function and overall mortality via the mTOR-ATF4 signaling pathway. We also introduce the possibility of gain-of-function furin variants (right panel) that could potentially mimic increased furin levels in promoting β-cell function and enhanced overall health. Further research is needed to fully understand the mechanisms involved and to develop targeted treatments and prevention strategies.

## Data Availability

No new data were created or analyzed in this study. Data sharing is not applicable to this article.
